# Supplementary material for: Inhibition of the FGF/FGFR System Induces Apoptosis in Lung Cancer Cells via c-Myc Downregulation and Oxidative Stress
Source: Int J Mol Sci. 2020 Dec 9;21(24):9376. doi: 10.3390/ijms21249376 (PMC7763353; doi:10.3390/ijms21249376)
Supplement: Supplementary file 1 [file ijms-21-09376-s001.pdf]

SUPPLEMENTARY FIGURES

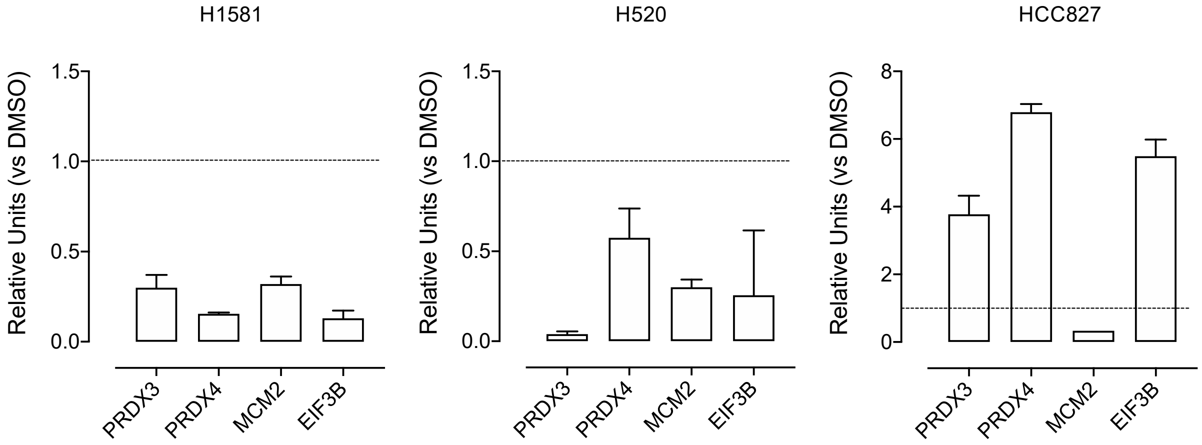

Fig. S1: RT-qPCR analysis of representative c-Myc target genes (as reported by Gene Set Enrichment Analysis [22]) in cells treated with 6 μM NSC12 for 24 hours. Dashed line indicates the vehicle/DMSO-treated cells. Data are mean ± SD.

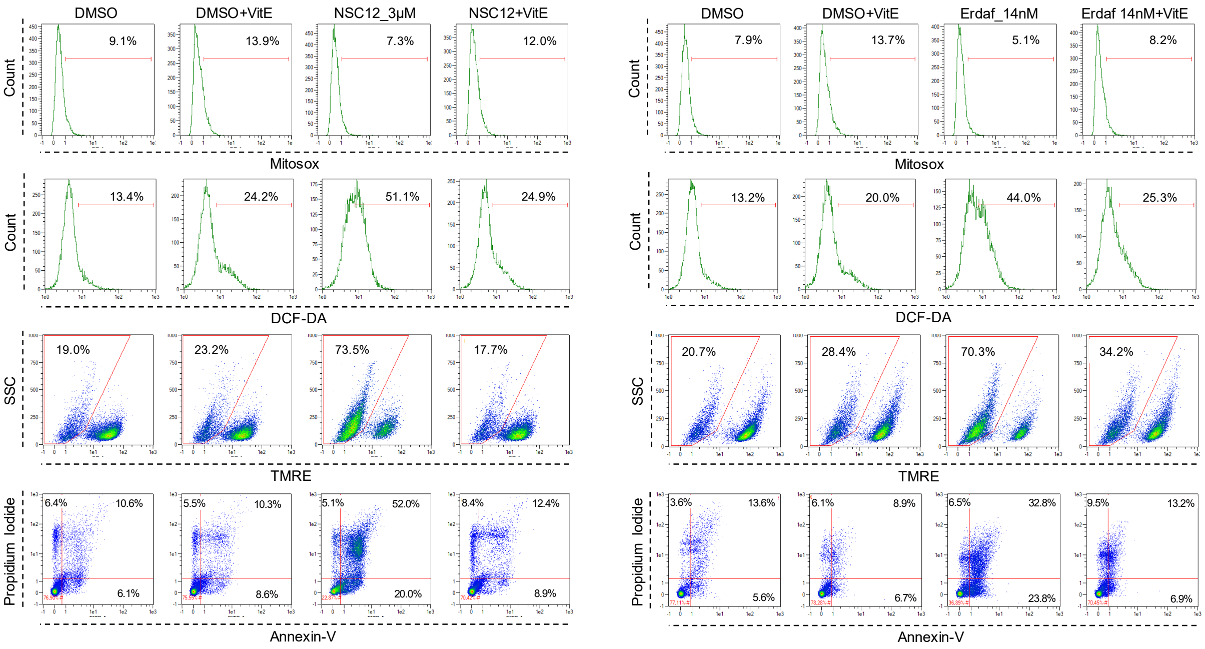

Fig. S2: Representative histograms (Mitosox and DCF-DA assays) and dot plots (TMRE and Apoptosis assays) from cytofluorimetric analysis of H1581 cells treated for 48 hours with 3.0 μM NSC12 or 14 nM Erdafitinib in presence or absence of 220 mM Vitamin E.

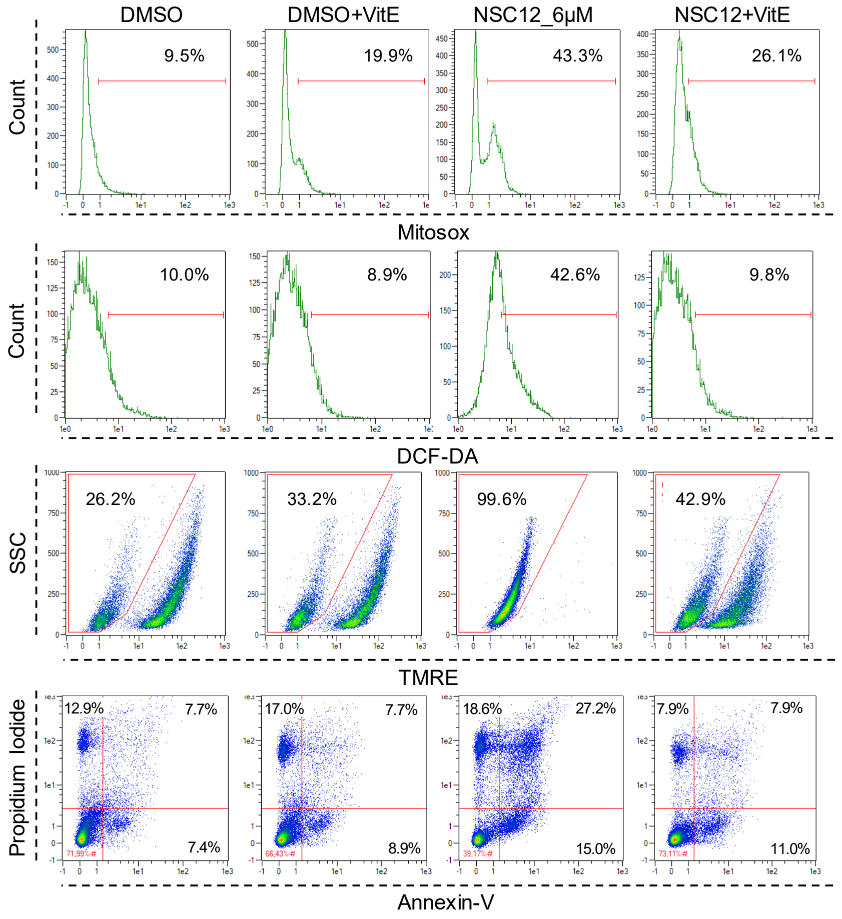

Fig. S3: Representative histograms (Mitosox and DCF-DA assays) and dot plots (TMRE and Apoptosis assays) from cytofluorimetric analysis of H520 cells treated for 48 hours with 6.0 μM NSC12 in presence or absence of 220 mM Vitamin E.

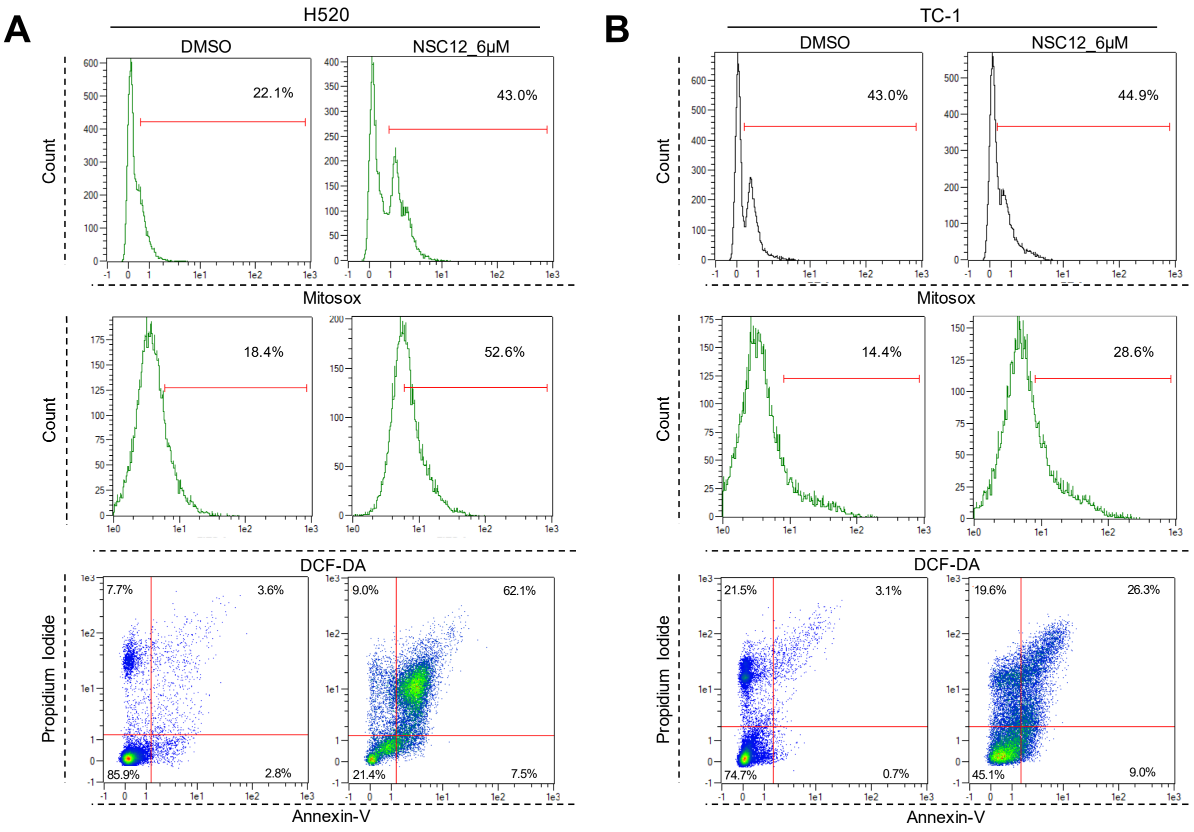

Fig. S4: FGF-independent TC-1 cells are less sensitive to FGF inhibition compared to FGF-dependent H520 cells. Mitochondrial (Mitosox) and cytoplasmic (DCF-DA) ROS production and apoptosis (Annexin-V/Propidium Iodide double staining) of H520 (**A**) and TC-1 (**B**) cells treated with 6.0 μM NSC12 for 48 hours.

	H1581	H520	HCC	TC-1
FGFR1	+++	+++	+	++
FGFR2	+++	++	++	-
FGFR3	++	++	+	-
FGFR4	++	++	++	-
FGF1	-	-	-	++
FGF2	++	++	++	++
FGF3	-	+++	-	-
FGF4	-	-	-	-
FGF5	-	-	++	-
FGF6	-	-	-	-
FGF7	-	-	-	-
FGF8	+	+	-	+
FGF9	-	-	-	++
FGF10	++	-	-	+++
FGF16	-	-	-	-
FGF17	++	++	+	+
FGF18	+	++	-	++
FGF19	++	+++	+	-
FGF20	-	-	-	-
FGF21	+	++	-	++
FGF22	-	-	-	-
FGF23	-	-	-	-

Table S1: Expression of FGFRs and FGFs (canonical and hormonal) were detected by qPCR in the indicated cell lines and relative levels were reported as highly expressed (+++), expressed (++), lowly expressed (+) or not expressed (-) based on ΔCt values for each gene compared to the housekeeping gene (GAPDH).
